# A gradual path to mortality

**DOI:** 10.7554/eLife.77749

**Published:** 2022-03-18

**Authors:** Na Yang, Payel Sen

**Affiliations:** 1 Laboratory of Genetics and Genomics, National Institute on Aging, National Institutes of Health Baltimore United States

**Keywords:** senescence, replicative senescence, epithelial to mesenchymal transition, Human

## Abstract

Many of the features associated with senescence appear steadily over time before cells stop dividing.

**Related research article** Chan M, Yuan H, Soifer I, Maile TM, Wang RY, Ireland A, O’Brien JJ, Goudeau J, Chan LJG, Vijay T, Freund A, Kenyon C, Bennett BD, McAllister FE, Kelley DR, Roy M, Cohen RL, Levinson AD, Botstein D, Hendrickson DG. 2022. Novel insights from a multiomics dissection of the Hayflick limit. *eLife* **11**:e70283. doi: 10.7554/elife.70283.

In 1961, Hayflick and Moorhead discovered that human fibroblast cells cultured in the laboratory could only divide a limited number of times, after which they stopped multiplying but remained metabolically active (Hayflick and Moorhead, 1961). It did not matter how the cells were cultured – whether they were repeatedly transferred, or ‘passaged’, into a fresh environment, or if their growth was interrupted by episodes of freezing – the number of divisions they could make was always finite. This state was termed replicative senescence and was found to occur in a range of cell types.

Further research revealed that senescence is caused by the shortening of caps, or ‘telomeres’, on the end of chromosomes (Bodnar et al., 1998). Every time a cell divides, its telomeres shrink until they reach a critical length that stops the cell from multiplying. Once senescent, cells display unique features such as expressing certain proteins, releasing inflammatory molecules, and loosening tightly packed regions of DNA known as heterochromatin (Hernandez-Segura et al., 2018). New evidence showed that senescence is induced by cell stress as well as successive divisions, and that the number of senescent cells increases as tissues age (van Deursen, 2014).

Despite almost 60 years of research, many questions still remain about senescence; for instance, what happens to cells as they transition in to the senescent state? How does their metabolism change during this shift, and do they take on a new cell identity? Now, in eLife, David Botstein, David Hendrickson and colleagues from Calico Life Sciences – including Michelle Chan as first author – report the results of experiments that exquisitely profile the roadmap cells take on their path to senescence (Chan et al., 2022).

The team used a new experimental design to survey the entire genome and repertoire of RNAs, proteins, and metabolites present in fibroblasts cultured in the laboratory. These patterns were traced over time as the cells grew until they stopped dividing. Chan et al. then used a range of control conditions to pinpoint which changes were specific to replicative senescence. This included repeating the experiment on cells growing at a similar density to senescent cultures, cells that had only been passaged a few times (and therefore unlikely to be senescent), cells that never become senescent, and cells made senescent by radiation-induced stress.

To begin with, Chan et al. measured the level of unique RNAs in single cells to investigate how the genes that fibroblasts expressed changed over time. The data revealed that RNAs known to be expressed in fully senescent cells progressively accumulate throughout the cell cycle. This suggests that senescent cells in vivo may be slowly amassing these features, but not yet expressing the classic biomarkers associated with the end-point of senescence, such as the beta-galactosidase enzyme. This may explain why previous studies found less than 20% of cells in old tissues exhibited these biomarkers, which has led researchers to question the role of senescence in aging (Biran et al., 2017; Idda et al., 2020). Chan et al. also found cells that had experienced replicative senescence (but not senescence induced by radiation) expressed genes related to a subtype of epithelial-to-mesenchymal transition (EMT). This process, which sees epithelial cells lining surfaces of the body lose their identity and become mesenchymal, is common in development and cancer.

Further experiments revealed that cells change how they use and generate energy as they progress towards replicative senescence. Similar metabolic changes have also been observed in models of EMT, further validating the connection between replicative senescence and this transition. Chan et al. discovered that the protein complex YAP1/TEAD1 and its target, the growth factor protein TGFβ2, drove this shift in energy. Applying a drug that stops YAP1 and TEAD1 from assembling (Wang et al., 2016) reduced the expression of signatures associated with mesenchymal cells and senescence.

Finally, Chan et al. identified another signature of replicative stress: the increased expression of Nicotinamide N-methyltransferase (NNMT). This enzyme catalyzes chemical reactions that ultimately prevent certain proteins from condensing DNA. It is possible that by reducing the activity of these proteins, NNMT is able to open up closed regions of the genome, which may explain the reported loss of heterochromatin in senescent cells (Yang and Sen, 2018).

The findings of Chan et al. suggest that cells gradually acquire a number of changes on the path to replicative senescence: they express different genes, rewire their metabolic reactions and take on a new identity similar to mesenchymal cells (Figure 1). Previous studies have shown that removing senescent cells can increase the health- and life-span of mice (Di Micco et al., 2021). Therefore, interventions that target these early changes could help improve the wellbeing of individuals by stopping the cascade of events that lead to replicative senescence.

**Figure 1.**
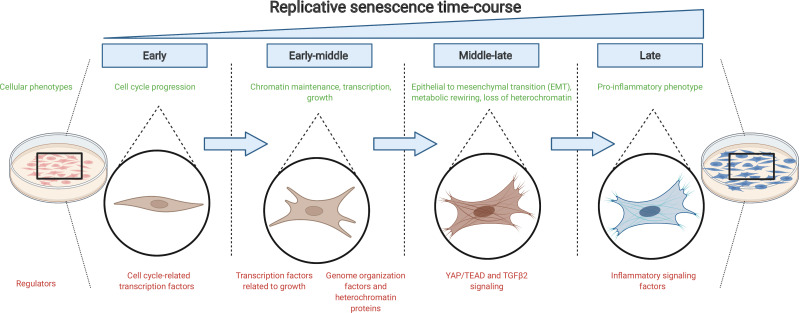
The journey to replicative senescence. Healthy cells cultured in the laboratory (left) will undergo continuous rounds of division until they reach a state of growth arrest called replicative senescence (right; shown in blue). To begin with (early and early-middle stages), cells express factors associated with cell cycle progression and growth as well as proteins that maintain chromatin stability. As they progress towards replicative senescence (middle-late stage), they undergo an epithelial-to-mesenchymal transition (EMT) and change their cell identity: this transition is driven by the growth factor protein TGFβ2 and the protein complex YAP1/TEAD1. As cells near replicative senescence (late stage), they exhibit a pro-inflammatory phenotype and secrete cytokines, in addition to expressing a beta-galactosidase enzyme which is detected by a reaction that turns cells blue.

Image credit: Figure created using BioRender.
